# Reshaping the immune landscape: next-generation microglia-targeted therapies for Alzheimer’s disease

**DOI:** 10.1186/s40659-026-00716-8

**Published:** 2026-07-10

**Authors:** Jun Wu, Jueying Zhao, Shen Chen, Fangyi Xu

**Affiliations:** 1 Department of Central Laboratory, Room 419, The Affiliated Huai’an No. 1 People’s Hospital of Nanjing Medical University, NO. 1 West Huanghe Street, Huai’an, 223300 Jiangsu China; 2 Department of Gynecology, Jiangwan Hospital of Hongkou District, Shanghai, 200434 China

**Keywords:** Alzheimer’s disease, Microglia, Immuno-targeted therapies, Metabolism-targeted therapies, Cell-based therapies, EV-based therapies

## Abstract

**Background:**

Alzheimer’s disease (AD) is a significant global health challenge characterized as a multifactorial neurodegenerative disorder, involving amyloid-β (Aβ) and Tau aggregation, neuroinflammation and progressive neuronal injury. While Amyloid-targeted therapies have achieved a breakthrough in prevention of Aβ aggregation, the strategies face notable limitations in achieving curative outcomes and management of amyloid-independent central nervous system (CNS) dysfunction. Consequently, targeting microglia, the central immune cells of the brain, has emerged as a promising strategy to enhance the specificity and efficacy of AD interventions.

**Main body:**

Accumulating evidence indicates microglial dysfunction is not a passive immune bystander of AD, but serves as a critical mechanistic nexus linking Aβ accumulation and AD symptomatic phenotype. This review critically examines the “next generation” of microglial therapeutics, moving beyond broad immunosuppression to precision phenotype modulation. We highlight breakthrough strategies in recent years including immune reconstitution, metabolic reprogramming, nanomaterial-mediated drug delivery, and the revolutionary potential of iPSC-derived microglia replacement. By elucidating the rationale underlying the specific strategies based on microglial biofunction and potential molecular mechanism in AD pathology, we provide an overview of current development of clinical trials and cutting-edge modalities aimed at restoring microglial homeostasis, affording an opportunity to alter the AD trajectory.

**Conclusion:**

This review aims to delineate the path from bench to bedside and propose promising pathways to overcome current bottlenecks in AD drug development.

## Introduction

With the acceleration of global population aging, neurodegenerative disorders, represented by Alzheimer's disease (AD), have emerged as a critical public health challenge threatening global well-being [1]. The median survival time following diagnosis ranges from merely 5 to 12 years [2]. Current standard-of-care therapies (cholinesterase inhibitors and NMDA receptor antagonists) provide only transient symptomatic relief. Although lecanemab and donanemab have marked important milestones by significantly slowing cognitive decline [3, 4], their clinical efficacy in halting cognitive decline remains modest. Consequently, there is an urgent need to develop innovative approaches that complement these amyloid-centric immunotherapies.

In this context, microglia, the brain’s resident innate immune cells, have garnered intense interest. They not only participate in Aβ phagocytosis but also express multiple AD risk-associated molecules, including TREM2, CD33, and APOE. Recent advances in multi-omics integration have underscored their pivotal role within the neuroimmune axis, highlighting their complex role in modulating AD pathology [5–8].

In this review, we critically evaluate the next generation of microglial-targeted therapeutics. Moving beyond simple immunosuppression, we synthesize emerging multidimensional modalities, ranging from gene editing and immunometabolic reprogramming to nanoparticle delivery and cell replacement therapies. By integrating these frontier approaches, we aim to delineate definitive pathways for precision modulation of the neuroimmune landscape, offering a promising and practical approach to circumvent current therapeutic bottlenecks in Alzheimer’s disease.

## Challenges of strategies targeting Aβ

In recent years, anti-Aβ antibodies such as lecanemab and donanemab have represented an important step forward in disease-modifying therapy, with positron emission tomography (PET) imaging confirming the effective clearance of cerebral Aβ plaques [9, 10]. However, this Aβ-centric strategy faces notable limitations. First, while classified as disease-modifying therapies, their clinical benefits remain modest; the progression of cognitive decline is merely slowed, not halted or reversed. Second, these therapies are associated with significant safety concerns, primarily Amyloid-Related Imaging Abnormalities (ARIA), which include edema (ARIA-E) and microhemorrhages (ARIA-H). Finally, even though triggered by Aβ, chronic neuroinflammation mediated by microglia has evolved into a self-perpetuating cycle independent of the initial Aβ trigger by the time overt symptoms manifest. Accordingly, current clinical guidance acknowledges the historical significance of these agents while emphasizing careful patient selection and individualized risk–benefit assessment. They emphasize a prudent assessment of the individual risk–benefit ratio and restrict use to selected early-stage patients. For patients unresponsive to or ineligible for antibody therapy, our therapeutic strategies should shift to downstream events.

## Microglia as a promising therapeutic target in AD

Microglia are the resident innate immune cells of the central nervous system (CNS). Although historically viewed as secondary responders to Aβ accumulation, recent genomic studies have repositioned microglia as central players in AD pathogenesis [11]. Genome-wide association studies (GWAS) reveal that the majority of risk genes for sporadic AD, including *TREM2*, *CD33*, *APOE*, the *MS4A* cluster, and *PLCG2*, are highly or exclusively expressed in microglia [12–15]. These findings provide compelling evidence that microglia are active drivers rather than passive bystanders in AD pathogenesis, catalyzing a paradigm shift from a strictly neurocentric view to one focused on neuro-immune interactions.

Mechanistically, genetic predispositions and pathological stress trigger a transition from a homeostatic state toward a disease-associated microglia (DAM) state. This state is characterized by the loss of signature markers like *P2RY12, TMEM119, and CX3CR1* [16, 17]. Initially, this transition is thought to be a protective response driven by the TREM2-APOE axis, upregulating genes involved in lipid metabolism and phagocytosis to clear cellular debris [14]. However, as the disease progresses and microglia are chronically exposed to persistent pathological Aβ and tau aggregates, the accumulation of cytotoxic debris further drives microglia toward a microglial neurodegenerative phenotype (MGnD), marked by the robust expression of *SPP1, CLEC7A, and LGALS3* [18]. In this late stage, microglial phagocytic capacity markedly declines, and they paradoxically fuel a self-amplifying cascade of neurotoxicity. They release chronic inflammatory mediators such as TNF-α and IL-1β, alongside high levels of reactive oxygen species (ROS) [19, 20]. These toxic mediators attack healthy synapses, disrupt neurotransmission, and ultimately induce neuronal death and AD progression. Damaged neurons generate more debris, further exacerbating microglial burden and dysfunction.

Importantly, this phenotypic transition extends well beyond AD pathology. Other neurodegenerative conditions, such as amyotrophic lateral sclerosis (ALS) and multiple sclerosis (MS), as well as natural aging, can also drive microglia to shift from a homeostatic state to a DAM or DAM-like phenotype. As disease progresses, these cells undergo a further transition from a DAM-like state to a maladaptive MGnD state [20–22]. Furthermore, microglial activation does not occur in isolation. Dysfunctional microglia release pro-inflammatory cytokines, such as TNF-α, IL-1α, and C1q, that can induce neurotoxic states in astrocytes (A1-like astrocytes) [23], thereby amplifying the neuroinflammatory cascade and accelerating synaptic pruning and neuronal loss.

The core strategy for microglia-targeted AD therapy seems to lie in precise modulation to restore their homeostatic protective functions, specifically efficient phagocytosis and neurotrophic support, while suppressing chronic inflammation. However, targeting microglia presents substantial challenges. These include stage-dependent microglial heterogeneity, potential off-target effects, and the limited ability of therapeutic agents to cross the blood–brain barrier. Recognizing these limitations is crucial for designing next-generation modalities or microglia-targeted antibodies.

## MG immuno-targeted therapies for AD

### Immuno-checkpoint targeted therapies

GWAS have fundamentally reshaped our understanding of sporadic AD (sAD) by revealing numerous risk variants associated with microglial biology [24, 25]. Notably, key risk genes such as *TREM2*, *APOE*, and the *MS4A* cluster are preferentially or exclusively expressed in microglia rather than neurons. This genetic insight underscores the potential of gene-targeted therapies as a precise modality to rectify microglial dysfunction.

**Fig. 1:**
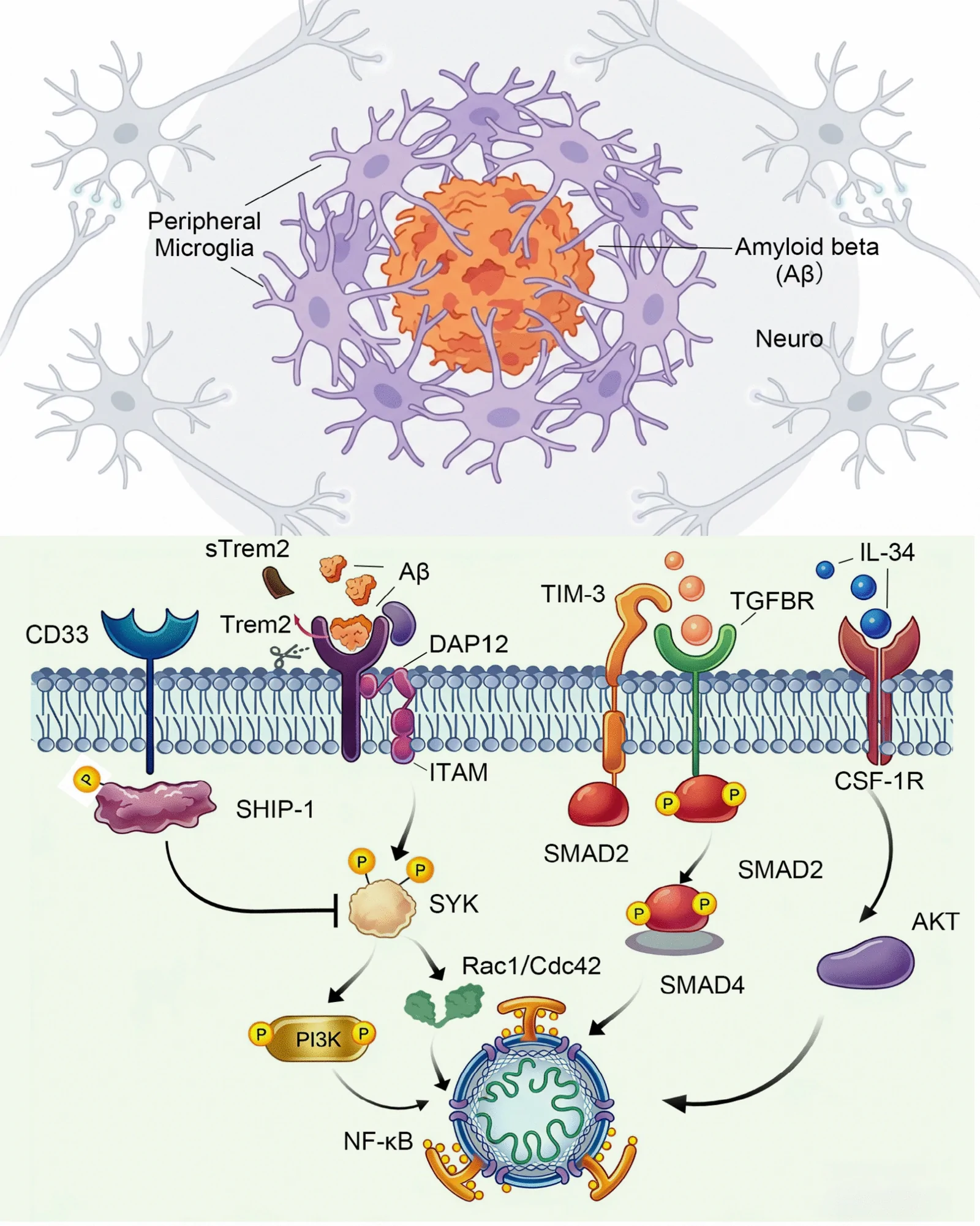
Molecular mechanisms driving microglia activation. The membrane-bound receptor TREM2 specifically senses ligands such as APOE-containing lipoproteins and Aβ. Ligand binding triggers receptor clustering and phosphorylation of immunoreceptor tyrosine-based activation motif (ITAM) within the cytoplasmic tail of DAP12 by Src-family kinases. Spleen tyrosine kinase (SYK) is recruited and then phosphorylated at the plasma membrane. SYK then activates multiple downstream effectors, like PI3K/AKT signals, to regulate the microglia function and metabolism. The TREM2 ectodomain is cleaved by ADAM10/ADAM17 to produce sTREM2, serving as an extracellular signaling molecule that feeds back on microglia. CD33 triggers SHIP-1 phosphorylation and functions in opposition to TREM2 through dampening SYK/PI3K-dependent activation. TIM-3 is upregulated by TGFβ signals and, in turn, triggers the SMAD2/4-TGFBR2 cascade to augment TGFβ signaling. Additionally, IL-34 and CSF-1 binding to CSF-1R maintain microglia survival via the PI3K/AKT pathway

*TREM2* encodes a cell-surface immunoreceptor that surveys the neural environment, sensing a diverse array of ligands, including lipoproteins and apolipoproteins. Upon ligand binding, TREM2 recruits the adaptor protein DAP12, initiating intracellular phosphorylation of SYK (Fig. 1). This signaling cascade is essential for maintaining microglial survival, metabolic fitness, inflammatory tuning, and phagocytic competency [12, 14]. In the context of AD pathology, TREM2 drives microglial clustering around amyloid plaques in part through its interaction with Aβ oligomers [26–28]. This clustering promotes plaque compaction and the formation of a neuroprotective barrier, thereby limiting the diffusion of toxic Aβ species and minimizing collateral neuritic dystrophy [29].

Beyond cell-surface signaling, TREM2 undergoes ectodomain shedding by α-secretases (ADAM10/17), releasing soluble TREM2 (sTREM2). sTREM2 retains biological activity, neutralizing Aβ neurotoxicity via a 'chaperone-like' mechanism [30, 31]. This protective role is corroborated by accumulating translational evidence. Cross-sectional human studies consistently show elevated CSF sTREM2 levels in AD patients [32–34], while longitudinal data indicate that higher baseline sTREM2 levels are associated with attenuated amyloid accumulation and slower cognitive decline [35]. Consistent with these clinical observations, preclinical TREM2 knockout models show impaired neurogenesis and exacerbated amyloid deposition, affirming its core neuroprotective function [29, 36, 37].

Building upon these biological discoveries, the initial therapeutic strategy focused on developing agonistic antibodies designed to stabilize TREM2, either by cross-linking the receptor or inhibiting ectodomain shedding. Driven by the preclinical success of the antibody 4D9, humanized agonist antibodies like AL002, VHB937, and VGN-740, were advanced into clinical development [38, 39]. However, while Phase I trials confirmed that these biologics were safe and achieved target engagement, Phase II outcomes have proved disappointing. Notably, neither AL002 nor VGN-740 met their primary efficacy endpoints for slowing cognitive decline, leading to their termination in 2024 and 2025 respectively [40]. These setbacks suggest that increasing surface expression via shedding inhibition may not be as effective as directly activating TREM2 signaling. Consequently, the field is pivoting toward small molecule agonists with superior physicochemical properties. At the forefront of this new wave is VG-3927, a first-in-class molecular glue designed to stabilize the TREM2-DAP12 interaction [41]. In Phase I trials, VG-3927 demonstrated high blood–brain barrier penetrance and dose-dependent modulation of sTREM2 with a favorable safety profile. Similarly, MNA-001, an orally bioavailable agonist, is currently under assessment in the Netherlands (CTIS2025-522368-34-00). Ultimately, the clinical failures of first-generation antibodies do not invalidate TREM2 as a target. The failure of these clinical trials to meet their anticipated endpoints prompts us to reflect on the potential underlying causes of this lack of efficacy. First, TREM2 plays distinct stage-dependent roles during the progression of AD. In the early stages of AD, TREM2 activation exerts a protective effect by promoting microglial phagocytosis of Aβ; however, in advanced pathological stages, TREM2 activation leads to microglial overactivation and aberrant synaptic pruning, which paradoxically exacerbates neuronal damage [42]. Second, soluble TREM2 (sTREM2), generated by the proteolytic shedding of TREM2, is associated with a decelerated AD disease course. Stimulation of TREM2 by AL002 triggers receptor internalization and degradation, accompanied by a significant reduction in cerebrospinal fluid (CSF) sTREM2 levels, potentially neutralizing the beneficial effects of the drug. Third, TREM2 agonists can induce adverse events such as amyloid-related imaging abnormalities (ARIA-E and ARIA-H). By activating perivascular macrophages, TREM2 signaling can provoke vascular inflammation, microvascular damage, and localized microhemorrhages [43]. Consequently, a substantial number of patients were unable to complete the requisite treatment course, further contributing to the negative clinical endpoints. Last but not least, microglial heterogeneity under AD pathological conditions and the functional complexity of TREM2 represent crucial factors. In early AD, microglia differentiate into the DAM subpopulation to enhance Aβ clearance and exert protective functions. In late-stage AD, however, microglia transition into an MGnD state, where their over activation aggravates neuronal injury. Systemic upregulation of TREM2 simultaneously activates both protective and pathogenic microglial subpopulations, thereby producing both beneficial and detrimental effects that may offset each other. Although the clinical efficacy was negative, these outcomes provide a critical theoretical basis for future microglia-targeted therapies and ARIA risk management [44].

Beyond TREM2, emerging targets implicated in Aβ clearance include the immune checkpoint CD33, as well as CD28, TMEM119, and TIM-3 (Table 1 and Fig. 1). Similarly, it is critical to recognize the temporal dynamism of receptor and checkpoint expression throughout the AD continuum. This necessitates a precision medicine approach tailored to the evolving microglial landscape, where interventions are timed specifically to disease progression. Recent studies suggest that AD progression can be monitored at the molecular level by integrating blood and CSF biomarkers, including Aβ, tau, and p-tau with PET imaging. In addition, transcriptomic profiling of CSF-derived cells and FAIMS-DIA-MS-based proteomic analysis provide further insight into dynamic molecular changes during disease progression [45, 46]. However, important limitations remain. In particular, CSF-derived myeloid cells may not fully reflect the phenotypic and functional states of parenchymal microglia, and the high cost and technical complexity of these approaches limit their broader application [47, 48]. Thus, although current technologies enable coordinated molecular-pathological monitoring, they are still insufficient for precisely defining microglial states across different stages of AD.

**Table 1:** Molecule of gene-targeted therapy for AD

Molecule	Source sample	Function	Mechanism	Therapeutic agent	Refs.
ACE	5 × FAD mice	ACE upregulated microglia to protect vulnerable neurons and excitatory synapses, and ameliorates learning and memory deficits	ACE enhances microglial phagocytosis of Aβ and promotes lysosomal trafficking, while also activating the TREM2/CLEC7A/SYK signaling cascade	–	[158]
TMEM119	5 × FAD, APP/PS1 and 3xTg mice	TMEM119 maintaining microglial homeostasis and improving cognitive deficits	TMEM119 binding Aβ oligomers and recruiting LRP1 to form a complex, leading to promote Aβ degradation through the lysosomal pathway	–	[159]
Cx43	APP/PS1 mouse	Ablation of microglial Cx43 hemichannels drive the shift of microglia status, suppress neurotoxicity, and prevent AD progression	Deletion or pharmacologic mimicry reprograms microglia to a protective phenotype, reduces neuroinflammation and toxicity, and ameliorates pathology, leading to cognitive improvement	TAT-Cx43@LNPs	[148]
ADGRG1	5 × FAD mice	ADGRG1 promotes a protective microglial state	Activating the transcription factor MYC that stimulates genes expression targeting cellular homeostasis, phagocytosis, and lysosomal functions	–	[160]
TIM-3 (*Havcr2*)	5 × FAD mice	Havcr2 knockout improve cognitive impairment and ameliorate Aβ deposition	TIM-3 binds to SMAD2 through its carboxy-terminal tail, leading to TGFBR-mediated SMAD2 phosphorylation and activation of downstream TGFβ cascade	–	[161]
Axl /Mer	APP/PS1 mouse and Human postmortem AD brain	Depletion of Axl and Mer leads to microglial dysfunction, impairing their ability to detect and phagocytose amyloid-beta plaques	Inducing Aβ plaque decoration by the TAM ligand Gas6 and its co-ligand phosphatidylserine	Gas6	[162]
PILRA	Human iMG and chimeric AD mice	*PILRA* knockout limits liptoxicity, improves mitochondrial stress, and inhibits inflammation	PILRA modulates microglial activity via two key mechanisms: it suppresses PPAR-mediated gene expression and lipid droplet formation, while activating a proinflammatory cascade through the STAT1 pathway	PILRA antibody	[163]
CD28	5 × FAD mice	PU.1^low^ CD28-expressing microglia subsets rescue neuroinflammation, preserve synapse density, and slow the progression of AD	Plaque induces downregulation of microglial PU.1 via the TREM2/CLEC7A-SYK–PLCγ2 axis, leading to a neuroprotective shift	–	[8]
MFE-2	Humans with AD and AD model mice	Genetic ablation of MFE-2 disrupts microglia homeostasis, accompanied by increased lipid deposition and elevated Mito chorial ROS production	MEF-2 deficiency leads to the accumulation of very long-chain fatty acids (VLCFAs) and arachidonic acid, triggering excessive ROS and producing cytokine storm	CKBA	[164]
GLP-1R	Patients with AD and APP23 mice	GLP-1R agonist increased AMPK activity in microglia, inhibiting neuroinflammation and promoting Aβ phagocytosis	GLP-1RAs enhanced CaMKK2–AMPK signaling, leading to reduced BACE1-mediated cleavage of amyloid precursor protein and Aβ generation	GLP-1R agonists (GLP-1RAs)	[88]
INPP5D	iPSC-derived human microglia	INPP5D disrupts autophagic flux, activates the NLRP3 inflammasome, promotes CASP1 cleavage and IL-1β/IL-18 secretion, and indirectly alters synaptic-related gene expression in neurons	INPP5D serve as a negative regulator of PI3K/AKT signaling leading to NLRP3-ASC-CASP1 inflammasome activation, with potential involvement of CLEC7A and PLA2G7 signaling	3AC	[165]
Piezo1	5 × FAD mice	Piezo1 senses the increased stiffness of Aβ fibril-containing brain tissues in AD; its deficiency exacerbates Aβ plaque burden, synaptic dysfunction, and cognitive decline	Piezo1 mediates extracellular Ca^2^⁺ influx upon sensing Aβ fibril stiffness and triggers actin cytoskeleton reorganization, thereby promoting microglial activation	Yoda1	[166]
HK2	5 × fad and AD patients	Deletion or pharmacological inhibition of HK2 specifically enhances microglial phagocytosis of Aβ, reduces amyloid plaque burden, attenuates cognitive impairment, and increases ATP production in microglia	HK2 inhibition suppresses glycolysis, downregulates glucose-6-phosphate and fructose-6-phosphate, which upregulates lipoprotein lipase expression, activates lipid metabolism to generate ATP by NADPH	3-BP	[167]
C5aR1	Arctic48 mouse model	C5a-C5aR1 signal activation initiate pro-inflammatory phenotype of microglia. Disrupting C5aR1 signal cascade inhibits diseased-related gene expression in microglia and improves cognitive performance in AD model	C5aR1 agonist PMX205 specifically inhibit DAM1 subsets shift via blocking MAPK/NFκB signaling pathway and remodels intercellular communication by enhancing neuroprotective pathways	PMX205	[168, 169]
PLCG2	5 × FAD	PLCG2 M28L variant increases AD risk by exacerbating amyloid plaque burden, impairing microglial Aβ phagocytosis and synaptic function, while the hypermorphic P522R variant reduces AD risk by enhancing plaque-responsive microglial phenotypes, and preserving cognitive and synaptic plasticity	PLCG2 is activated by BTK-mediated phosphorylation, and regulates calcium-dependent transcription factors; its variants reshaping microglial transcriptional programs that govern inflammatory responses, phagocytosis, and lipid metabolism, and directing the transition to protective or detrimental plaque-responsive microglial subpopulations	–	[15]
VCAM1	APP/PS1 mice and AD patients	VCAM1 promotes Aβ chemotaxis in microglia and improves microglia clearance of Aβ	IL-33–ST2 and VCAM1–ApoE pathways induces Aβ-directed migration in microglia	IL-33	[170]
CD33	hES derived microglia	HuM195 enhances the uptake of Aβ42 by downregulating CD33 and promoting IL-33 expression, thereby reprogramming microglia toward a protective state	HuM195 transiently drives CD33 internalization and phosphorylation, leading to CD33 degradation and thus activates microglia to increase Aβ clearance capabilities	HuM195 (Lintuzumab)	[13]
C1q	*APPswe/PS1deltaE9* mice	Inhibit microglia overactivation and ameliorate cognitive impairment	Curbing complement-mediated microglial synaptic engulfment and preserving synapse architecture	pioglitazone	[109, 171, 172]
TREM2	5 × FAD	TREM2 deletion or R47H variant impair microglial clustering around Aβ plaques, phagocytosis, and acquisition of the DAM phenotype, exacerbating Aβ burden, neuronal damage, and cognitive decline	The DAP12-TREM2 complex activates SYK-dependent signaling to trigger the PI3K-AKT-GSK3β-mTOR pathway, sustaining microglial metabolic fitness and DAM differentiation	–	[36, 173]
APOE	5 × FAD and iMG	APOE3 enhances microglial activation, increases plaque-associated microglia, reduces amyloid pathology and neuritic toxicity, and improves cognitive/synaptic function, while APOE4 restricts these protective responses, promotes lipid droplet accumulation, and impairs microglial surveillance, phagocytosis, and injury responsiveness	APOE3 drives the transcriptional signature of activated response microglia by upregulating antigen presentation and interferon pathways, and optimizing lipid metabolism; in contrast, APOE4 suppresses complement and lysosomal pathways, activates mTOR/eIF2 stress signaling, and disrupts lipid homeostasis	–	[99, 100]

### Microglial immunopathway-targeted therapies for AD

**Fig. 2:**
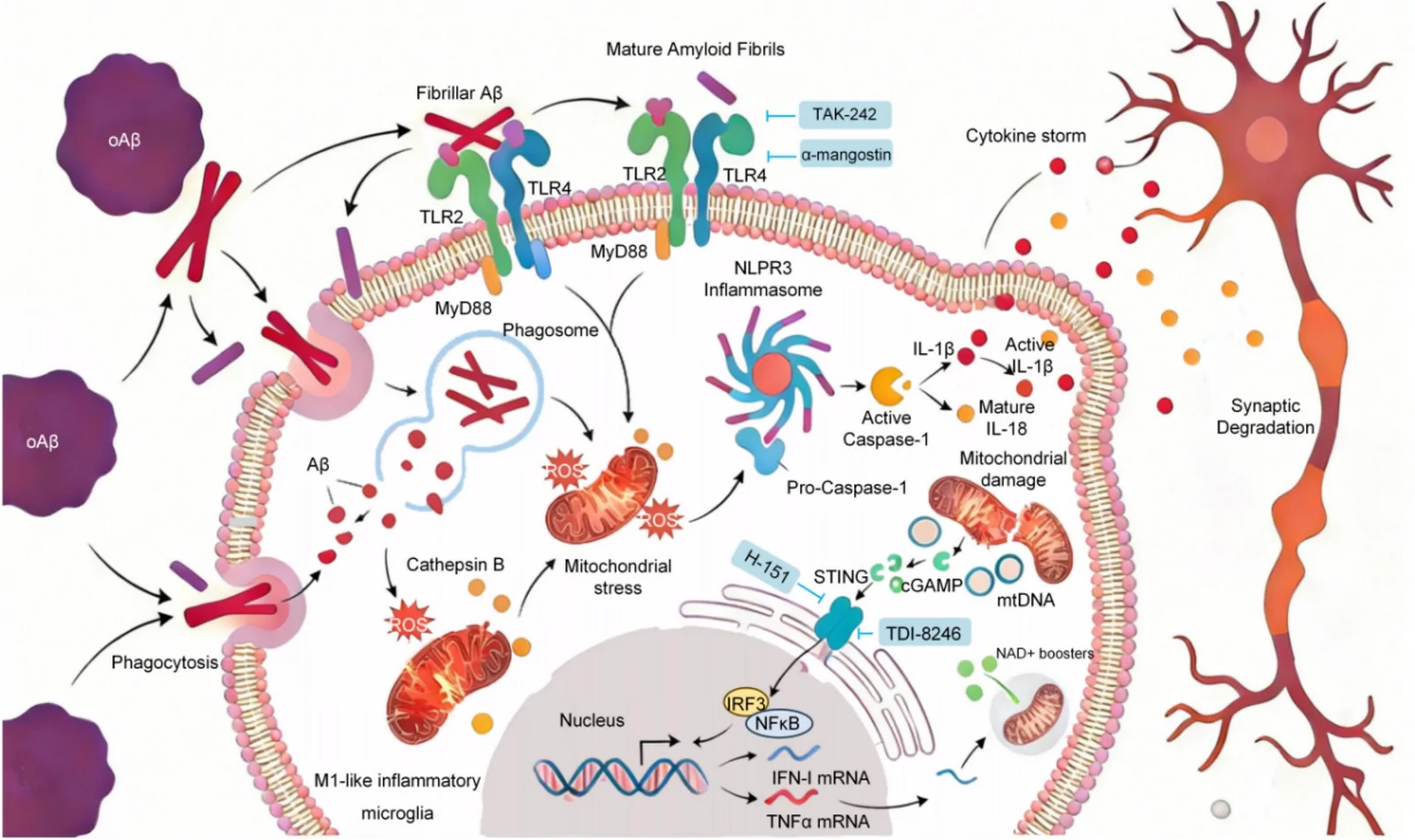
Inflammatory cascades trigger microglia shifting to M1-like phenotype. Fibrillar Aβ activates microglial TLR2 and TLR4, initiating MyD88-dependent signaling and priming microglia. TLR2/4-driven inflammatory signaling and Aβ-induced cellular stress promote mitochondrial dysfunction, leading to enhanced mitochondrial ROS (mtROS) production; mtROS act as key triggers that couple mitochondrial stress to NLRP3 activation. The resulting assembly of the NLRP3 inflammasome enables caspase-1 activation, proteolytic maturation of IL-1β and IL-18. Excessive secretion of IL-1β, IL-18, TNF-α and IL-6 drives synaptic dysfunction, neuronal injury, and progressive neurodegeneration by promoting excitotoxicity, disrupting neuronal homeostasis, and sustaining a self-reinforcing neuroinflammatory microenvironment. In parallel, damaged mitochondria lead to mtDNA leakage, which triggers activation of cGAS–STING signaling, resulting in IRF3 nuclear translocation and upregulation of IFN-I. Selected pharmacological inhibitors targeting TLR4 signaling, such as TAK-242 and α-mangostin, or targeting cGAS-STING pathway, like H-151 and TDI-8246 were developed to alleviate microglia pro-inflammatory activation

In the microenvironment of Alzheimer’s disease (AD), the accumulation of Aβ plaques and tau pathology precipitates a maladaptive shift in microglia toward a hyperactivated, neurotoxic phenotype. This maladaptive activation state is characterized by the chronic secretion of pro-inflammatory cytokines (TNF-α, IL-1β) and chemokines (such as CXCL2), creating a localized inflammatory milieu that accelerates aberrant synaptic pruning and neuronal loss. Given that broad immunosuppression risks abolishing beneficial microglial functions (such as phagocytosis), the therapeutic paradigm has evolved toward the precise modulation of key signaling nodes [49–52] (Fig. 2).

#### NLRP3 inflammasome

Nucleotide-binding oligomerization domain (NOD)-like receptors (NLRs), which control host defense and complement surface receptor signaling, are found in cytosolic multimeric protein complexes called inflammasomes. Upon detecting pathogen-associated or damage-associated molecular patterns (PAMPs/DAMPs), NLRs assemble into inflammasomes to initiate inflammatory cascades. In the CNS, the NLRP3 inflammasome is a key factor in AD immunopathology because it is primarily assembled in microglia [53]. Mechanistically, the microglial internalization of fibrillar Aβ (fAβ) induces lysosomal destabilization and the subsequent leakage of cathepsin B into the cytosol [54]. Thereafter, cathepsin B in turn further promotes NLRP3 activation by recruiting the adaptor protein ASC to form a scaffold that activates caspase-1, which cleaves pro-IL-1β and pro-IL-18 into their active forms. This sustained inflammatory cascade not only perpetuates neuroinflammation but also drives pyroptosis [53–58]. Distinct from this lysosomal pathway, oligomeric Aβ (oAβ) activates NLRP3 via mitochondrial dysfunction and reactive oxygen species (ROS) generation, processes often coupled with membrane perturbation and potassium efflux [59, 60].

Human post-mortem analyses corroborate this mechanism, revealing that inflammasome activation, marked by caspase-1 upregulation, is detectable in early AD and mild cognitive impairment (MCI) [61]. Consistent with these findings, genetic deletion of *Nlrp3* or *Asc* in transgenic mouse models ameliorates cognitive deficits, reduces Aβ accumulation, and attenuates tau hyperphosphorylation [53, 62]. Pharmacologically, small-molecule NLRP3 inhibitors, such as MCC950 and CRID3, have demonstrated robust preclinical efficacy [63–65]. Translating these findings, several candidates are now progressing through clinical pipelines. Notably, usnoflast (Phase II for amyotrophic lateral sclerosis; NCT05981040) and NT-0796 (Phase II for Parkinson’s disease) exhibit significant potential for treating neurodegenerative disorders. Crucially, unlike compounds restricted to peripheral indications, CNS-targeted inhibitors must possess sufficient blood–brain barrier (BBB) penetrance. Emlenoflast is a BBB-penetrant NLRP3 inhibitor with potent anti-inflammatory activity, evidenced by the improvement of Parkinson's pathology in preclinical PET-MRI studies [66]. Although Roche discontinued Emlenoflast for undisclosed reasons, this discontinuation has spurred the industry to develop next-generation NLRP3 inhibitors with superior potency and to investigate synergistic therapeutic strategies [67].

Minocycline, a second-generation tetracycline that readily crosses the BBB, has shown neuroprotective effects in preclinical AD models by suppressing microglial overactivation, reducing tau hyperphosphorylation, and limiting Aβ deposition. In animal studies, it alleviated cognitive deficits and neuropathological changes induced by Aβ or lipopolysaccharide [68, 69]. However, this preclinical promise has not translated well clinically: randomized trials such as MADE found no significant cognitive benefit in patients with mild-to-moderate AD [70], and although minocycline reduced microglial activation in TBI patients, it was also associated with increased neurodegenerative markers and worsened axonal injury [71]. Overall, given the complexity of AD, a single anti-inflammatory agent is unlikely to be sufficient.

#### cGAS–STING pathway

**Fig. 3:**
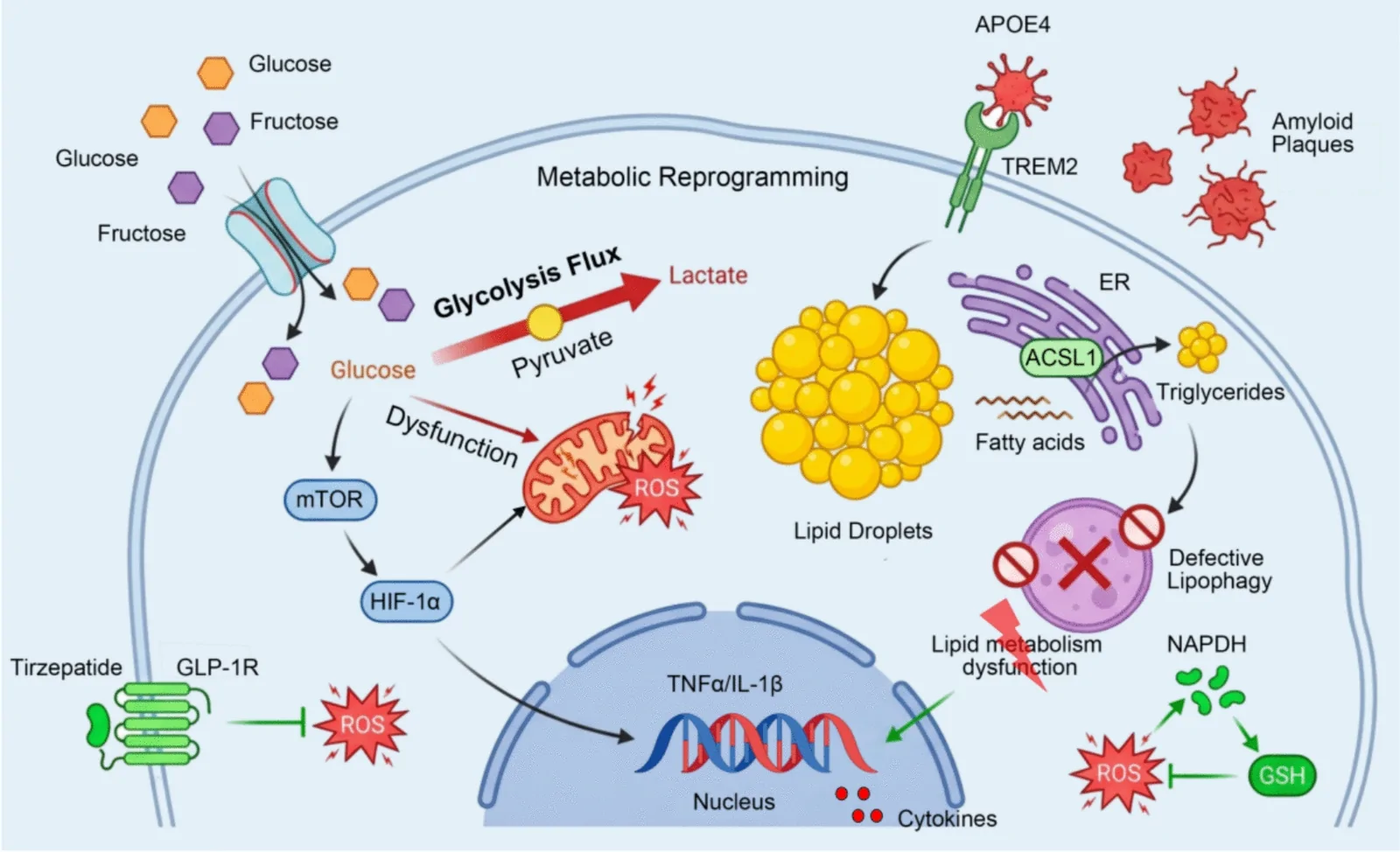
AD stress induces microglia metabolic reprogramming. Aβ exposure triggers mitochondrial dysfunction and activates the mTOR–HIFα axis in DAM, shifting cellular metabolism from oxidative phosphorylation to glycolysis. This metabolic stress is further exacerbated by high concentrations of fructose, which worsen mitochondrial damage and ROS production. Furthermore, microglia inundated with lipid-rich debris from amyloid–lipoprotein complexes convert excess fatty acids into triglycerides via ACSL1 for storage in lipid droplets (LDs). The resulting LD accumulation impairs protective lipophagy, leading to oxidative stress overload. This excessive ROS disrupts GSH-mediated redox homeostasis, driving microglia toward a pro-inflammatory state with enhanced secretion of TNFα and IL-1β. Notably, Tirzepatide (a GLP-1R agonist) has emerged as a promising therapeutic agent capable of counteracting this oxidative stress by effectively downregulating ROS levels

The cyclic GMP–AMP synthase (cGAS)–stimulator of interferon genes (STING) pathway serves as a critical cytosolic surveillance mechanism in microglia [72, 73]. In AD, Aβ deposition and tau hyperphosphorylation trigger oxidative stress and mitochondrial damage, precipitating the leakage of mitochondrial DNA (mtDNA) into the cytosol. This mtDNA is sensed by cGAS, which catalyzes the synthesis of cGAMP to activate STING on the endoplasmic reticulum. Subsequent signaling induces the nuclear translocation of IRF3, driving type I interferon expression (Fig. 3). Concurrently, NF-κB activation upregulates TNF-α, IL-6, and chemokines. This chronic inflammatory state directly compromises neuronal integrity, impairs microglial Aβ clearance, and accelerates cellular senescence [74].

Human cohort studies have revealed dynamic fluctuations in CSF mtDNA levels, characterized by a significant decrease in preclinical AD—a pattern distinct from the sharp increase observed in frontotemporal lobar degeneration [75]. Other studies have also confirmed elevated mtDNA levels in patients with MCI and symptomatic AD [76]. Consistent with these findings, elevated interferon levels corroborate the hyperactivation of the cGAS–STING axis. In murine models (5 × FAD and P301S), *Cgas* knockout has been shown to attenuate neuroinflammation and preserve cognitive function [77].

Current therapeutic strategies targeting this signaling cascade fall into two distinct approaches. The first involves reducing upstream mtDNA leakage by employing NAD+ activators, such as nicotinamide riboside, to boost mitophagy [78]. The second focuses on the direct inhibition of core pathway components using cGAS inhibitors, such as H-151 and TDI-8246 [72, 73, 77]. Currently, over 30 cGAS-targeting inhibitors are in development, including IMSB301 and VENT03. However, several major challenges remain unresolved, limiting their translation into clinical benefit.

Microglial activation in AD is highly time-dependent: early protective responses eventually shift to a chronic pro-inflammatory phenotype. Unfortunately, clinical anti-inflammatory interventions typically commence during mid-to-late stages, completely missing the optimal therapeutic window. Furthermore, because inflammatory pathways (such as NLRP3, cGAS-STING) are also broadly expressed in neurons and astrocytes [79, 80], non-targeted small molecules inevitably trigger off-target effects. Moreover, single-target inhibitors cannot halt the complex neuroinflammatory cascade. Extruded mtDNA simultaneously activates parallel pathways (cGAS-STING, NLRP3, TLR9, and AIM2) [81]; thus, blocking a single node cannot prevent compensatory activation. In late-stage AD particularly, MGnD, the disease-associated population, exhibit prolonged mPTP opening and continuous mtDNA leakage [73]. Inhibitors targeting downstream mediators fail to repair this upstream mitochondrial dysfunction or neutralize the massive ROS production that sustains the inflammatory microenvironment. Pharmacologically, poor BBB penetrability and a lack of microglia-specific targeting further prevent these drugs from reaching effective intracellular concentrations. Crucially, multi-omics analyses reveal that human microglial heterogeneity do not conform to the oversimplified M1/M2 dichotomy. Single-cell RNA sequencing analyses have shown that activated microglia in the human brain occupy transitional states, co-expressing pro- and anti-inflammatory markers [82, 83]. One of the most important reasons for decades of translational failure is that “anti-inflammatory targets” identified through the binary M1/M2 models in rodents may completely lack regulatory efficacy, or paradoxically exacerbate pathology within the highly heterogeneous and complex landscape of human AD.

## Microglial metabolism-targeted therapies for AD

### Glucose metabolism

Microglia exhibit remarkable plasticity, adapting their phenotype through metabolic reprogramming to meet bioenergetic demands. Upon acute Aβ exposure, microglia trigger the mTOR–HIF-1α pathway, shifting metabolism from oxidative phosphorylation (OXPHOS) to glycolysis to fuel phagocytosis [84, 85]. However, in the chronically stressed environment of AD, persistent Aβ accumulation disrupts this normal metabolic regulation, causing oxidative stress and excessive ROS production (Fig. 3). This global dysfunction precipitates a sustained shift toward glycolysis, accompanied by the production of biosynthetic intermediates required for inflammatory mediators [84, 86]. Concurrently, oxidative stress affects the pentose phosphate pathway (PPP) by upregulating the rate-limiting enzyme glucose-6-phosphate dehydrogenase (G6PD), leading to increased NADPH generation. While NADPH is essential for antioxidant defense, its overproduction paradoxically exacerbates mitochondrial ROS and promotes NO synthesis, establishing a feed-forward cycle of metabolic dysfunction and inflammation. Beyond glucose, fructose metabolism has also emerged as a critical regulator of microglial function. The fructose transporter GLUT5, predominantly expressed by microglia in the CNS, is upregulated in AD mouse models and patient samples [84]. Indeed, high-fructose diets have been shown to exacerbate disease progression by driving aberrant microglial activation and mitochondrial defects [87].

Recent pharmacological advances highlight the potential of targeting these metabolic pathways. Originally indicated for type 2 diabetes (T2D), Glucagon-like peptide-1 receptor (GLP-1R) agonists like semaglutide have demonstrated significant neuroprotective properties. Emerging evidence indicates that these agonists cross the BBB to activate the energy sensor AMPK, thereby reducing Aβ production and enhancing microglial clearance [88]. Clinically, this therapeutic potential is supported by epidemiological data showing that semaglutide use significantly lowers dementia risk in T2D patients [89]. Currently, semaglutide is undergoing Phase III clinical trials for AD (NCT04777396, NCT04777409) [90], while other GLP-1R agonists, such as tirzepatide and exendin-4, have shown encouraging preclinical outcomes. Another strategic avenue involves restoring microglial redox balance. Currently, supplementation with glutathione precursors, including glycine and cysteine, is being evaluated in Phase II clinical trials. These nutrient interventions aim to boost endogenous GSH synthesis, attenuate microglial oxidative stress, or revive mitochondrial function. Furthermore, orally available small molecules such as SUL-238, which stimulate mitochondrial complex I and IV activities, have demonstrated safety, tolerability, and CNS penetration in Phase I trials in elderly volunteers. These agents also represent promising candidates for metabolic reprogramming.

### Lipid metabolism

In the AD microenvironment, microglia are inundated with lipid-rich debris derived from amyloid–lipoprotein complexes and degenerating neuronal membranes. When the lysosomal degradation capacity is saturated by the chronic phagocytosis of this debris, free fatty acids and cholesterol are released into the cytosol. Consequently, excess fatty acids are shuttled to the endoplasmic reticulum, where long-chain acyl-CoA synthetase 1 (ACSL1) and diacylglycerol O-acyltransferase 2 catalyze their esterification for storage in lipid droplets (LDs) [91, 92]. This accumulation is further exacerbated by AD-associated impairments in lipophagy and dysfunction of the lipid transporter ABCA1. Pathologically, LDs physically compress organelles and drive a shift to a pro-inflammatory phenotype, releasing ROS and cytokines (like IL-6, CCL2) that activate neighboring cells and perpetuate neurodegeneration [93] (Fig. 3).

GWAS have identified key lipid regulators as AD risk loci, including *APOE, ABCA7, CLU, SORL1,* and *TREM2*. The *APOE* ε4 allele represents the most potent risk factor, increasing susceptibility 9–12 fold [94, 95]. Mechanistically, APOE4 impairs microglial function via multiple pathways. Firstly, APOE4 exhibits a significantly lower binding affinity for TREM2 than APOE3, thereby hindering TREM2-mediated phagocytosis of Aβ and tau [14, 96]. Secondly, APOE4 restricts lipid efflux and the clearance of proinflammatory cytokines (IL-1β, TNF) and complement factors, promoting plaque aggregation [97]. Thirdly, it upregulates *ACSL1*, predisposing microglia to a lipid droplet-accumulating phenotype [92, 93, 98]. These pathological effects are corroborated by in vivo models: *APOE4* knock-in mice show enhanced MGnD characteristics and impaired clearance of apoptotic neurons and Aβ. Conversely, microglia-specific ablation of *APOE4* reduces plaque burden in APP/PS1 mice and mitigates tau pathology in P301S models [99, 100]. These findings are further supported by human post-mortem analyses showing that APOE4 carriers exhibit a lower density of activated microglia surrounding plaques [101].

Consequently, research has intensified on dissecting how APOE regulates microglial metabolism, polarization, and phagocytosis, positioning it as a viable candidate for combinatorial immunotherapy [92, 93, 101–105]. Indeed, single-cell sequencing and spatial proteomics analyses of brain tissues from patients treated with anti-Aβ immunotherapy (such as AN1792, lecanemab) indicate that APOE is a crucial modulator of microglial-mediated clearance, with its expression positively correlating with therapeutic efficacy [97]. Emerging APOE-targeted interventions include antisense oligonucleotides (ASOs) for selective silencing (Phase I/II) and C-terminal mimetic peptides (CS-6253; Phase I). Furthermore, advanced gene therapy strategies, such as adeno-associated virus (AAV)-mediated APOE2 gene replacement therapy, have demonstrated efficacy in counteracting APOE4-associated toxicity and conferring neuroprotection [106, 107]. Beyond APOE, peroxisome proliferator-activated receptor gamma (PPARγ) is considered a pivotal target for restoring lipid homeostasis in microglia and alleviating AD pathology. The potential of this strategy is evidenced by AD mouse models and clinical trials using its specific agonists, such as pioglitazone, T3D-959 and genistein [108–111].

Notably, agonists developed using murine models often demonstrate poor translatability in human clinical trials, primarily due to the significant species-specific metabolic divergence. While activated mouse microglia undergo a pronounced Warburg effect characterized by a glycolytic shift, human-derived microglia predominantly rely on fatty acid oxidation (FAO) with a negligible glycolytic response [112]. This fundamental metabolic difference explains why conventional glycolytic inhibitors, such as 2-DG, are largely ineffective in humans. Furthermore, regional microglial heterogeneity is a critical factor. Hippocampal microglia exhibit higher basal activation and a pro-inflammatory metabolic profile, rendering them highly sensitive to glycolytic/HIF-1α modulators [113]. In contrast, cortical microglia maintain a homeostatic surveillance state and are largely non-responsive to the same metabolic interventions. Additionally, clinical evidence indicates that metabolic agents (like GLP-1R agonists, metformin, or ketone supplements) offer only marginal benefits in MCI or early AD [114]. In advanced stages, cerebral metabolic damage becomes irreversible, and metabolic improvements frequently disconnect from clinical cognitive gains. This suggests that metabolic drugs alone are insufficient to break the Aβ-tau pathological cascade or halt functional decline. Consequently, while metabolic modulators may not serve as monotherapies for AD, they hold substantial promise as adjunct or supportive therapies to alleviate neuroinflammation, mitochondrial dysfunction, and lipid dysregulation [115]. Early intervention during the preclinical or MCI stages to block the pathological cascade may prove more effective than anti-Aβ or anti-tau strategies, positioning metabolic modulation as a superior approach for early prevention and intervention.

Taken together, while microglia-targeted therapies demonstrate immense potential, their translation from bench to bedside is impeded by significant scientific, technical, and clinical bottlenecks. This translational gap primarily stems from three major challenges. The first is the critical issue of timing. Microglia undergo significant changes very early in the disease process. By the time patients are treated in the mid-to-late stages of AD, these microglial alterations are often irreversible [116]. At this point, simply tweaking downstream metabolism or inflammatory pathways is largely ineffective. Similar to anti-Aβ therapies, microglial modulation would likely be most effective in the prodromal or preclinical stages, well before irreversible neuronal damage occurs. However, this early-intervention strategy requires precise screening and the ability to track microglial activation states in vivo. Unfortunately, current imaging techniques, such as TSPO-PET, lack the resolution to distinguish between specific protective or detrimental microglial phenotypes. Furthermore, the high plasticity of microglia implies that any therapeutic reprogramming might only be transient [117]. Without simultaneously addressing the underlying Aβ and tau pathologies, the reprogrammed cells are highly prone to reverting to a dysregulated state under continuous pathological stress. Second, relying heavily on mouse models introduces critical blind spots due to species differences. A promising drug target identified in rodents may actually play a very minor role in the human brain microenvironment. This issue is further complicated by the fact that microglia behave differently depending on their specific brain region, making a “one-size-fits-all” approach ineffective. Third, there is the persistent physical challenge of drug delivery. Many inhibitors do not cross the BBB efficiently, resulting in sub-therapeutic intracranial drug concentrations. Even when drugs do reach the brain, they often lack specificity. Given the functional homology between microglia, perivascular macrophages, and other brain-resident macrophages, non-selective drugs frequently trigger off-target side effects that compromise overall therapeutic benefits [118]. Thus, identifying surface markers that are truly exclusive to microglia remains an ongoing challenge for achieving precision treatment.

To overcome these dead ends, the field is developing toward more precise and personalized technologies. For example, microglial cell-replacement therapies are being explored to bypass the issues of intrinsic cell heterogeneity altogether, while nanoparticle-based delivery systems are actively designed to shuttle drugs past the BBB and deliver them explicitly to microglia.

## Cell-based therapies: microglial replacement and iPSC transplantation

Microglial replacement therapy (MRT) represents a disease-modifying strategy designed to reconstitute the cerebral immune microenvironment. The fundamental premise is to replace chronically dysfunctional and exhausted microglia with healthy, functional cells, thereby restoring homeostasis and facilitating the clearance of pathological substrates.

Early interventions utilized selective CSF-1R inhibitors, such as PLX3397 (pexidartinib), PLX5622, and GW2580, to eliminate resident microglia. Although these agents achieved near-complete ablation, microglia rapidly repopulated the CNS within one week of inhibitor withdrawal. During this repopulation phase, plaque reduction proved transient and was frequently followed by a rebound exceeding control level, indicating that depletion alone is insufficient for sustained therapeutic effect [119, 120]. Nevertheless, CSF-1R inhibition effectively creates a transient “microglia-free” niche, establishing a critical conditioning regimen for the subsequent adoptive transfer of exogenous therapeutic microglia. Regarding donor sources, bone marrow-derived stem cells (BM-SCs) possess the capacity to cross the BBB and differentiate into microglia-like cells within the CNS [121]. Furthermore, the technologies of embryonic stem cell (ESC) and human-induced pluripotent stem cell (hiPSC) provide a renewable, standardized, and functionally validated source of transplantable microglia [122, 123]. Recent protocols utilizing serum-free, chemically defined induction systems provide convenience to clinical translation, which meet Good Manufacturing Practice (GMP) standards and stringent FDA guidelines.

**Fig. 4:**
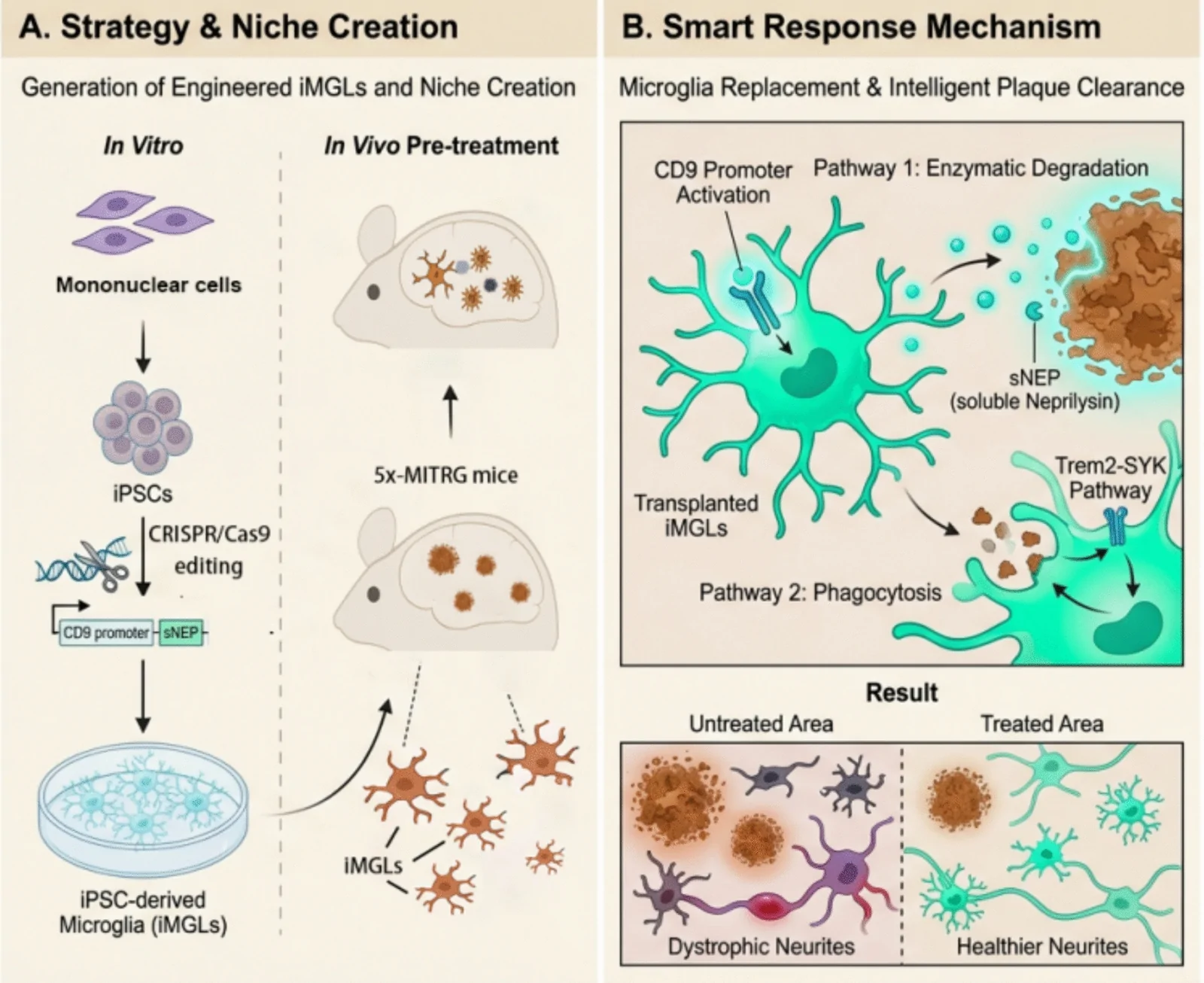
iMGs are promising candidates for cell therapy. iPSC-derived microglia (iMG) were engineered via CRISPR to express the Aβ-degrading enzyme neprilysin (NEP) under the control of the disease-associated microglia-specific CD9 promoter. In AD mouse models, the NEP-expressing iMG ameliorated pathology by reducing both soluble/insoluble Aβ loads and toxic oligomers, leading to the preservation of synaptic integrity and neuronal density. Mechanically, CD9 activates the TREM2-SYK signaling axis to potentiate phagocytosis. Furthermore, engraftment of iMGs into microglia-depleted mice (5 × MITRG mice), maximizing therapeutic efficacy in plaque-dense regions such as the subiculum. Importantly, these engineered cells possess the capacity to cross the BBB to access the CNS parenchyma

Beyond simple cellular substitution, MRT can actively remodel the pathological microenvironment. In a pivotal study, Yoo et al. transplanted circulation-derived myeloid cells (CDMCs) from *Trem2*^+/+^ donors into *Trem2*^−/−^;5 × FAD mice. This intervention inhibited Itgax, Ch25h, and Lpl expression in the DAM, while reactivating TREM2-SYK-mediated phagocytic signaling. Consequently, the treatment reduced amyloid plaque burden and attenuated LAMP1^+^ dystrophic neurites [124]. Genetic engineering has further refined MRT precision. For instance, iPSC-derived microglia (iMG) modified by CRISPR/Cas9 have been employed to place neprilysin (NEP) expression under the control of the *CD9* promoter. Since CD9 is selectively upregulated in the amyloid niche, this strategy confines NEP expression to plaque-associated regions and spares healthy parenchyma. This "sense-and-response" mechanism enabled in situ plaque degradation and significantly slowed AD progression in vivo [124] (Fig. 4).

Reliable protocols for microglial replacement have now been established, including whole-brain replacement via allogeneic bone marrow transplantation (mrBMT) or peripheral blood mononuclear cell infusion (mrPB), as well as region-specific replacement via direct injection (mrMT) [125–127]. These approaches have been evaluated in eight patients with adult-onset leukoencephalopathy with axonal spheroids and pigmented glia. After a 24-month follow-up, treatment reconstituted normal microglial populations, restored CSF-1R signaling, and significantly attenuated disease progression without serious adverse events [128].

In addition to direct cellular replacement, therapeutic strategies that harness the paracrine functions of low-immunogenicity human stem cells to remodel the central nervous system microenvironment are also of substantial clinical value. Exosomes secreted by bone marrow mesenchymal stem cells (BMSCs) have been shown to stimulate BDNF expression in hippocampal neurons, suppress microglial activation and the release of inflammatory cytokines, and thereby ameliorate AD-related pathology [129]. Moreover, BMSCs secrete bioactive factors such as TIMP2 and VEGF, which inhibit matrix metalloproteinase (MMP)-mediated shedding of the TIE2 receptor, reduce circulating soluble TIE2 (sTIE2) levels, improve vascular function, and attenuate chronic inflammation [130]. In 2025, a Phase II trial of Laromestrocel (BMSCs) in 49 patients with mild AD reported promising efficacy. A single intravenous injection reduced the rate of hippocampal atrophy by 61.9% and preserved total brain volume by 48.4%. Clinically, Montreal Cognitive Assessment (MoCA) scores improved by 0.9 points, an effect equivalent to reversing 6–8 months of cognitive decline. Moreover, the high-dose cohort (four injections) exhibited a 5.3-point improvement in Alzheimer’s Disease Cooperative Study-Activities of Daily Living (ADCS-ADL) scores. Notably, no amyloid-related imaging abnormalities (ARIA) were observed [131]. As of 2025, there are 35 clinical trials based on stem-cell interventions for AD registered on ClinicalTrials.gov, highlighting the robust global interest in this treatment method. Inspired by the functions of stem cell-derived exosomes, the enrichment and application of these vesicles as therapeutic agents or drug delivery carriers has emerged as an important direction in the development of cell-based therapies.

## Exosomes and engineered nanovesicles as novel carriers

Extracellular vesicles (EVs) are nanoscale (30–200 nm) lipid bilayer-enclosed vesicles actively secreted by cells. They mediate intercellular communication by transporting bioactive cargos—including proteins, lipids, and nucleic acids—from donor to recipient cells. As therapeutic platforms, EVs possess key advantages including low immunogenicity, high biocompatibility, natural ability to cross the BBB, and the potential for selective cell targeting.

### Therapeutic potential of natural EVs

Naturally derived EVs have demonstrated regenerative and neuroprotective potential. Mesenchymal stem cell–derived EVs (MSC-EVs) attenuate neuroinflammation and promote neural regeneration in multiple CNS disease models [132–134]. In AD mouse models, intranasal or systemic delivery of MSC-EVs reduces both Aβ and tau pathological burden, with improvements in cognition. Mechanistically, these effects are attributed to EV cargo—such as anti-inflammatory microRNAs and neurotrophic proteins—transferred to microglia, thereby reprogramming microglia from a pro-inflammatory M1-like phenotype to an anti-inflammatory, repair-promoting M2-like phenotype [132, 135, 136].

Similarly, human-induced pluripotent stem cell–derived neural stem cell EVs (hiPSC-NSC-EVs) administered to 5 × FAD mice are rapidly internalized by microglia, particularly plaque-associated microglia (PAM). This uptake downregulates NPR3-mediated inflammatory signaling and interferon-γ gene expression, attenuates hippocampal astrocytic hypertrophy, and reduces both Aβ plaque burden and phosphorylated tau (p-tau) levels. These molecular and cellular changes ultimately lead to improvements in cognition and emotional behavior [137]. In parallel, plasma-derived small EVs (sEVs) carrying microRNAs such as miR-144-3p, miR-149-5p, and miR-455-3p have been shown to stimulate PGC-1α expression, thereby enhancing mitochondrial energy metabolism and cognitive function in AD models [138].

### Plant-derived EVs (PDEVs)

PDEVs offer distinct translational advantages, including cost-effective scalability, the potential for oral administration (thereby minimizing injection burden), and the absence of zoonotic pathogen risks. Mechanistically, PDEVs exert anti-inflammatory effects through multiple complementary mechanisms. Enriched in plant polyphenols or anti-oxidative lipids, they can suppress inflammatory signaling by inhibiting phosphorylation upstream of TLR4 and at the IKK/IκB node, thereby blocking NF-κB nuclear translocation and reducing transcription of TNF-α, IL-6, and IL-1β. PDEVs also attenuate excessive MAPK signaling by decreasing phosphorylation of p38, JNK, and ERK [139]. In addition, PDEVs from sources such as ginger and ginseng have been reported to inhibit NLRP3 inflammasome assembly, ASC speck formation, and caspase-1 activation, thereby reducing the release of IL-1β and IL-18 [140, 141]. Beyond direct pathway inhibition, PDEVs may reshape the inflammatory microenvironment by promoting a shift of microglia from a pro-inflammatory M1-like phenotype toward a more anti-inflammatory M2-like state, increasing IL-10 and TGF-β expression [142, 143]. They may also modulate host cells through plant-derived miRNAs. Vesicle-encapsulated miRNAs can be stably delivered into host cells and may post-transcriptionally repress inflammatory genes, including targets such as TNFα, IL-6, and IL-17, through interaction with their 3′UTRs [144, 145]. Furthermore, our previous work revealed that oat-derived nanovesicles (OatN) possess intrinsic BBB permeability and can successfully target microglia with approximately 85% efficiency, thereby significantly alleviating inflammatory stress responses. Collectively, PDEVs expand the repertoire of precision delivery platforms for AD therapy.

### Engineering strategies to enhance EV therapeutics

**Fig. 5:**
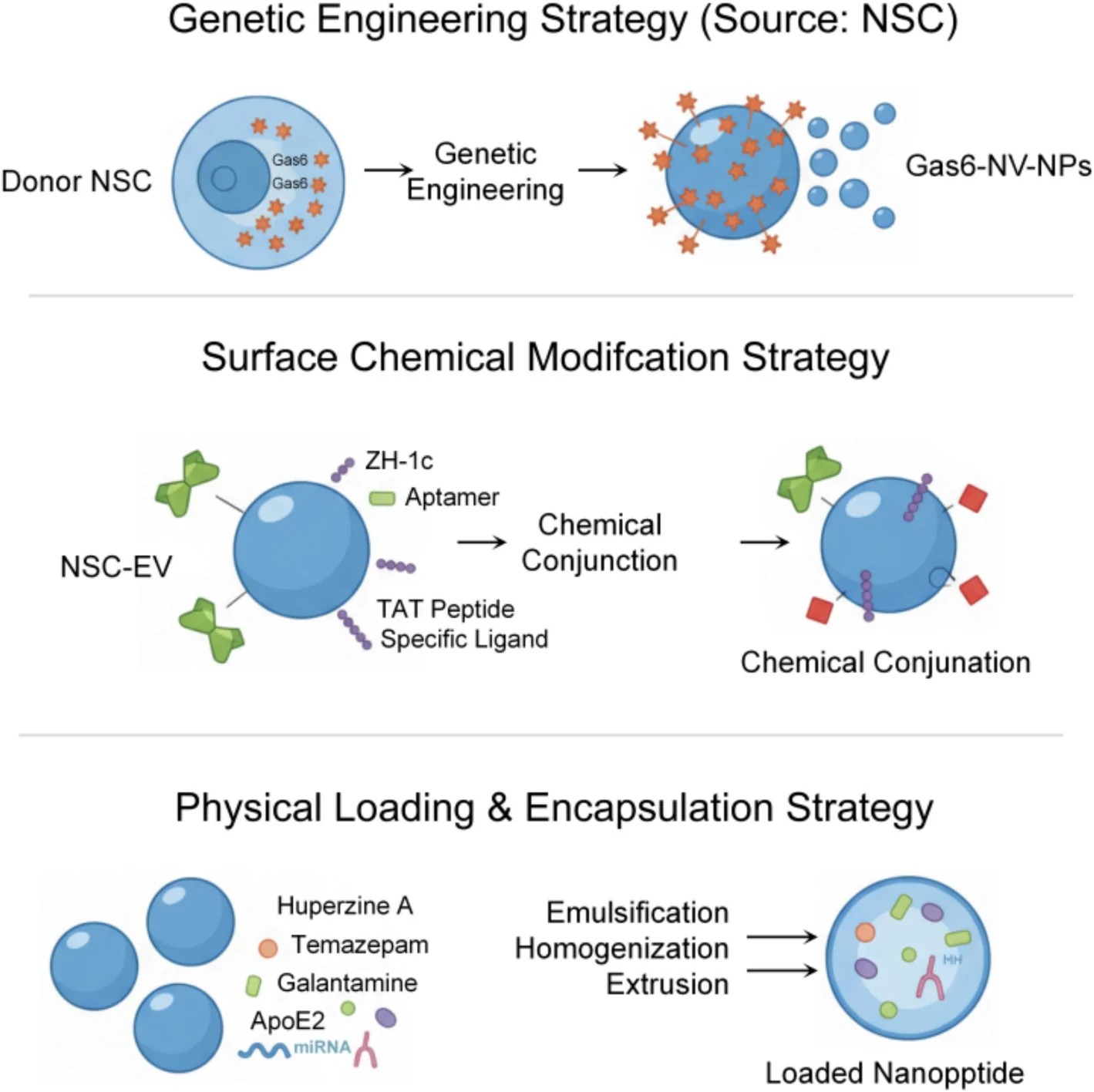
Engineering EVs targeting AD. Engineering approaches to enhance CNS delivery and therapeutic efficacy. Firstly, exogenous expression of Gas6 in neuro stem cell enriches Gas6 on the surface of NSC derived nanoparticles (Gas6-NV-NPs), leading to enhanced phagocytosis by microglia and increased cellular uptake. Secondly, conjugating EVs with specific ligand, such as TAT peptide or ZH-1c amtamer, facilitates transport across the blood–brain barrier through interaction with lipid bilayer or membrane receptor such as CD64. Thirdly, loading the nanoparticles with therapeutic agents and restructuring EVs via extrusion or emulsification techniques improve the drug efficiency. These methods prolong the drug half-life and ensure targeted release

Despite the aforementioned advantages, natural extracellular vesicles (EVs) face inherent limitations, including insufficient targeting specificity, limited therapeutic potency, and compositional heterogeneity. To circumvent these hurdles, bioengineering strategies have been developed to augment therapeutic efficacy by employing surface functionalization and cargo loading techniques. Genetic engineering of donor cells can significantly enhance EV targeting efficiency; for instance, the overexpression of growth arrest-specific 6 (Gas6) on the surface of neural stem cell (NSC)-derived nanovesicles (Gas6-NV-NPs) facilitates specific uptake by microglia [146]. Alternatively, chemical conjugation allows the attachment of specific ligands or cell-penetrating peptides (such as TAT) to the EV surface [147, 148]. A notable example involves conjugating the ZH-1c aptamer to NSC-EVs, which specifically binds to CD64 on activated microglia and improves delivery efficiency [149]. Furthermore, the vesicular architecture of these nanocarriers enables high-density cargo enrichment, prolonged circulation half-life, and spatiotemporally controlled release. Depending on the hydrophilicity and charge of the therapeutic agents, encapsulation techniques such as emulsification, homogenization, and extrusion have been successfully employed to load diverse payloads—ranging from small molecules (huperzine A, temazepam, and galantamine) to macromolecules (ApoE2 peptides and miRNAs) [150–154]. By addressing critical bottlenecks, such as optimizing BBB penetration, improving cargo stability, and minimizing off-target effects, engineered nanosystems have become promising candidates for Alzheimer’s disease (AD) therapy (Fig. 5).

In a clinical context, an open-label Phase I/II trial (NCT04388982) evaluated the safety and efficacy of intranasally administered exosomes derived from human mesenchymal stem cells. Over a 12-week period, the treatment was well-tolerated with no reported adverse events and biochemical safety profiles within normal limits. Notably, the medium-dose group exhibited reduced hippocampal atrophy and significant cognitive improvement, evidenced by a 3.98-point decrease in ADAS-cog scores [155]. Despite these advancements, no EV-based therapeutics have yet received FDA approval. Clinical translation currently faces two primary hurdles: (1) the lack of standardized manufacturing protocols, characterized by low scalability, batch-to-batch variability, and inconsistent loading efficiency; and (2) insufficient long-term safety data, specifically concerning systemic accumulation and potential neurotoxicity [156, 157]. However, given the paradigm-shifting success of lipid nanoparticle (LNP)-encapsulated mRNA vaccines against SARS-CoV-2, the prospect of EV-based therapies remains highly promising.

## Conclusion and future perspectives

The therapeutic landscape of AD is undergoing a profound paradigm shift, moving from the long-established amyloid-centric era toward a new age of multi-target precision medicine. Within this transformative framework, robust genetic evidence positions microglia as the central nexus linking Aβ pathology, tauopathy, and neurodegeneration, establishing them as one of the most promising therapeutic targets.

Consequently, the adoption of combinatorial or sequential regimens represents an inevitable evolution of standard treatment. Future strategies will likely transcend monotherapies; instead, they will strategically integrate agents with distinct mechanisms adapted to specific disease stages. For example, in the early stage, high-potency anti-Aβ antibodies (e.g., lecanemab or donanemab) could be employed to rapidly lower cerebral Aβ burden, thereby relieving microglia of ongoing pathological stress. Subsequently or concurrently, TREM2 agonists or immunomodulators could be administered to mitigate immune hyperactivation and restore microglial homeostasis. It is anticipated that such a “clear-and-repair” approach will yield synergistic efficacy beyond simple additive effects, maximizing the potential to slow or halt disease progression.

Furthermore, cutting-edge platforms like gene therapy, cell replacement, and engineered extracellular vesicles provide novel tools for circumventing current therapeutic barriers. Although facing challenges related to delivery, safety, scalability, and cost, these technologies represent transformative modalities capable of modulating biological processes at their source. As they develop and their safety profiles are thoroughly validated, these technologies have the potential to offer groundbreaking treatment options for patients with particular molecular subtypes or those who are not responding to traditional interventions.

## Data Availability

Not applicable.

## Abbreviations

AD: Alzheimer’s disease
Aβ: Amyloid-β
CSF: Cerebrospinal fluid
CSF-1R: Colony-stimulating factor 1 receptor
DAM: Disease-associated microglia
GLP-1R: Glucagon-like peptide-1 receptor
GWAS: Genome-wide association studies
iPSC: Induced pluripotent stem cell
LDAM: Lipid droplet-accumulating microglia
MGnD: Microglia of neurodegenerative phenotype
MRT: Microglial replacement therapy
OXPHOS: Oxidative phosphorylation
PPP: Pentose phosphate pathway
ROS: Reactive oxygen species
TLR: Toll-like receptor
TNFα: Tumor necrosis factor-alpha
EV: Extracellular vesicle
ACE: Angiotensin I converting enzyme
Cx43: Connexin 43
ADGRG1: Adhesion G protein-coupled receptor G
MFE2: Peroxisomal multifunctional enzyme type 2
INPP5D: Inositol polyphosphate-5-phosphatase D
HK2: Hexokinase 2
PI3K: Phosphatidylinositol 3-kinase
TREM2: Triggering receptor expressed on myeloid cells
CD33: Siglec-3
APOE: Apolipoprotein E
MS4A: Membrane-spanning 4-domains subfamily A
PLCG2: Phospholipase C gamma 2
ABCA7: ATP-binding cassette subfamily A member 7
CLU: Clusterin
SORL1: Sortilin-related receptor 1
PGC-1α: Peroxisome proliferator-activated receptor gamma coactivator 1-alpha
